# Differences in the immunoglobulin gene repertoires of IgG versus IgA multiple myeloma allude to distinct immunopathogenetic trajectories

**DOI:** 10.3389/fonc.2023.1123029

**Published:** 2023-02-08

**Authors:** Glykeria Gkoliou, Andreas Agathangelidis, Georgos Karakatsoulis, Chrysavgi Lalayanni, Apostolia Papalexandri, Alejandro Medina, Elisa Genuardi, Katerina Chlichlia, Evdoxia Hatjiharissi, Maria Papaioannou, Evangelos Terpos, Cristina Jimenez, Ioanna Sakellari, Simone Ferrero, Marco Ladetto, Ramon Garcia Sanz, Chrysoula Belessi, Kostas Stamatopoulos

**Affiliations:** ^1^ Institute of Applied Biosciences, Centre for Research and Technology Hellas, Thessaloniki, Greece; ^2^ Department of Molecular Biology and Genetics, Democritus University of Thrace, Alexandroupoli, Greece; ^3^ Department of Biology, School of Science, National and Kapodistrian University of Athens, Athens, Greece; ^4^ Department of Mathematics, School of Sciences, University of Ioannina, Ioannina, Greece; ^5^ Hematology Department and HCT Unit, G. Papanikolaou Hospital, Thessaloniki, Greece; ^6^ Hematology Department, University Hospital of Salamanca, Salamanca, Spain; ^7^ Department of Molecular Biotechnologies and Health Sciences, Hematology Division, University of Turin, Turin, Italy; ^8^ First Department of Internal Medicine, AHEPA University Hospital, Aristotle University of Thessaloniki, Thessaloniki, Greece; ^9^ Department of Clinical Therapeutics, National and Kapodistrian University of Athens, Athens, Greece; ^10^ Hematology Department, Nikea General Hospital, Piraeus, Greece; ^11^ Department of Molecular Medicine and Surgery, Karolinska Institute, Stockholm, Sweden

**Keywords:** multiple myeloma, immunogenetics, immunoglobulin isotypes, immunoglobulin a, immunoglobulin g, immunoglobulin gene repertoire, somatic hypermutation (SHM), n-glycosylation

## Abstract

The analysis of the immunogenetic background of multiple myeloma (MM) has proven key to understanding disease ontogeny. However, limited information is available regarding the immunoglobulin (IG) gene repertoire in MM cases carrying different heavy chain isotypes. Here, we studied the IG gene repertoire in a series of 523 MM patients, of whom 165 and 358 belonged to the IgA and IgG MM groups, respectively. IGHV3 subgroup genes predominated in both groups. However, at the individual gene level, significant (p<0.05) differences were identified regarding IGHV3-21 (frequent in IgG MM) and IGHV5-51 (frequent in IgA MM). Moreover, biased pairings were identified between certain IGHV genes and IGHD genes in IgA versus IgG MM. Turning to the imprints of somatic hypermutation (SHM), the bulk of rearrangements (IgA: 90.9%, IgG: 87.4%) were heavily mutated [exhibiting an IGHV germline identity (GI) <95%]. SHM topology analysis disclosed distinct patterns in IgA MM versus IgG MM cases expressing B cell receptor IG encoded by the same IGHV gene: the most pronounced examples concerned the IGHV3-23, IGHV3-30 and IGHV3-9 genes. Furthermore, differential SHM targeting was also identified between IgA MM versus IgG MM, particularly in cases utilizing certain IGHV genes, alluding to functional selection. Altogether, our detailed immunogenetic evaluation in the largest to-date series of IgA and IgG MM patients reveals certain distinct features in the IGH gene repertoires and SHM. These findings suggest distinct immune trajectories for IgA versus IgG MM, further underlining the role of external drive in the natural history of MM.

## Introduction

Multiple myeloma (MM) is a malignancy characterized by the accumulation of terminally differentiated clonal plasma cells (PCs) in the bone marrow (BM). It accounts for 10% of hematologic malignancies and 0.8% of all cancer diagnoses, respectively, with 130,000 new cases every year worldwide (1). The main feature of MM is the secretion of a specific monoclonal immunoglobulin (IG) by the malignant PCs which can be detected in the serum and/or the urine of patients with MM. MM is classified into different types depending on the isotype of the IG heavy and light chain. The predominant myeloma type is IgG (52%), followed by IgA (21%), light chain (16%), bi-clonal (2%), and IgM (0.5%), while IgD and IgE myelomas are rather infrequent (2).

Immunogenetic analysis of MM has proven key to understanding disease ontogenesis. Indeed, relevant studies have reported: (i) clonal relationship between switch variants expressed by the BM PCs and myeloma progenitors in the BM and blood (3, 4); (ii) virtual absence of the inherently autoreactive IGHV4-34 gene (5–7); and, (iii) patterns of somatic hypermutation (SHM) suggestive of a post-germinal center (GC) derivation (8, 9). Yet, limited evidence exists about the architecture of the IG gene repertoire in different types of MM characterized by the expression of different heavy chain isotypes, in particular IgA versus IgG. This is relevant for the ontogeny of MM, especially in the context of conflicting evidence regarding the immunogenetic profiles of IgA versus IgG memory B cells in non neoplastic conditions. Indeed, a higher SHM burden has been reported for CD27^+^IgA^+^ versus CD27^+^IgG^+^ normal memory B cells, perhaps due to the distinct microenvironment of the respective immune responses, especially given that IgA class switching occurs mostly in mucosa-associated lymphoid tissues (10). On the other hand, studies in IgA and IgG expressing B cells in different settings (11–13) have found an overall similar burden of SHM; finally, BM IgG PC may display higher SHM rates compared to circulating IgG memory B cells (14). At the clinical level, patients with IgA MM exhibit a higher incidence of t(4;14) (40% in IgA versus 13% in IgG), shorter progression-free survival (PFS) and worse overall survival (OS) compared to patients with IgG MM (15, 16).

Here, we investigated potential differences in the immunogenetic profiles of IgA versus IgG MM in a large multi-institutional cohort. We report distinct profiles particularly regarding the individual frequencies of particular IGH genes, the topology and molecular features of SHM and the characteristics of the heavy variable complementarity determining region 3 (VH CDR3). These findings allude to different antigen exposure trajectories and/or affinity maturation processes for MM subtypes, i.e. IgA and IgG MM, further underscoring the key role of microenvironmental interactions in the pathogenesis of MM.

## Materials and methods

### Study group

All patients were diagnosed with MM following the IMWG criteria (17). Collected samples derived from collaborating Institutions in Greece (n=176), Italy (n=72) and Spain (n=201) and the IMGT/LIGM-DB public database (18) (n=74). The sequence datasets from the Italian and Spanish groups have been reported previously (references (19) and (20), respectively). The study was approved by the local Ethics Review Committees of each participating Institution and was conducted in accordance with the Declaration of Helsinki.

### Sample collection and sequencing analysis of IGHV-IGHD-IGHJ gene rearrangements

BM aspirates were collected from 449 patients with MM with a PC infiltration of at least 20%. Available immunogenetic data for the remaining 74 cases were retrieved from the IMGT/LIGM-DB public database (18). On the basis of serum immunoelectrophoresis/immunoblot analysis, 165 and 358 cases expressed clonotypic IG gene rearrangements belonging to the IgA and IgG isotypes, respectively.

BM mononuclear cells (BMMCs) were isolated by Ficoll-Hypaque density centrifugation. Genomic DNA (gDNA) extraction, total cellular RNA isolation and cDNA synthesis were performed as previously described (7, 21, 22). IGHV-IGHD-IGHJ gene rearrangements were PCR-amplified using Leader and IGHJ primers as previously described (7). PCR products were subjected to bidirectional Sanger sequencing.

### IGHV-IGHD-IGHJ gene rearrangement sequence analysis

IGHV-IGHD-IGHJ gene rearrangement sequences were annotated with the use of the IMGT/V-QUEST tool (23, 24). Only productive rearrangements were subjected to further analysis. IGHV, IGHD, and IGHJ gene usage, SHM molecular features and topology, and the characteristics (amino acid length and composition) of the VH CDR3 were extracted for each sequence.

IGHV-IGHD-IGHJ gene rearrangements were analyzed for VH CDR3 stereotypy through the use of our validated bioinformatics pipeline (25). In detail, a set of immunogenetic criteria were applied on the clustering process: (i) utilization of IGHV genes originating from the same phylogenetic clan, (ii) ≥ 50% amino acid identity and ≥ 70% similarity of the VH CDR3, (iii) same VH CDR3 length and, (iv) same offset of the common amino acid pattern.

The NetNglyc tool was used for the prediction of N-Glycosylation sites across the IGHV-IGHD-IGHJ gene rearrangement sequences, based on the application of artificial neural networks for the examination of the sequence context of Asn-Xaa-Ser/Thr sequons (26).

### Statistical analysis and data visualization tools

Descriptive statistics for qualitative variables included counts and frequency distributions. To examine potential differences in terms of immunogenetic characteristics between the two patient groups (IgA MM versus IgG MM), the chi-square test and Fisher’s exact test were applied. For all comparisons a significance level of α=0.05 was set. All statistical analyses were performed in R v.4.1.1.

Data visualization was performed in the R environment, with the open-source data visualization framework RawGraphs (https://rawgraphs.io/) and the Circos software package (http://mkweb.bcgsc.ca/tableviewer/).

## Results

### The IGHV gene repertoires of IgA and IgG MM patients display both shared but also unique characteristics

All 7 IGHV gene subgroups were represented in our cohort. In specific, IGHV3 predominated at cohort level (279/523 rearrangements; 53.3%), followed by IGHV4 (96/523 rearrangements; 18.4%) (**Supplemental Table 1**). At the individual IGHV gene level, 47 distinct IGHV genes were identified. Seven IGHV genes were defined as frequent (individual frequency ≥4%), collectively accounting for 41.9% of the total repertoire: IGHV3-30, IGHV3-23, IGHV3-9, IGHV3-33, IGHV4-59, IGHV2-5 and IGHV1-69 (**Supplemental Table 2**).

**Figure 1:**
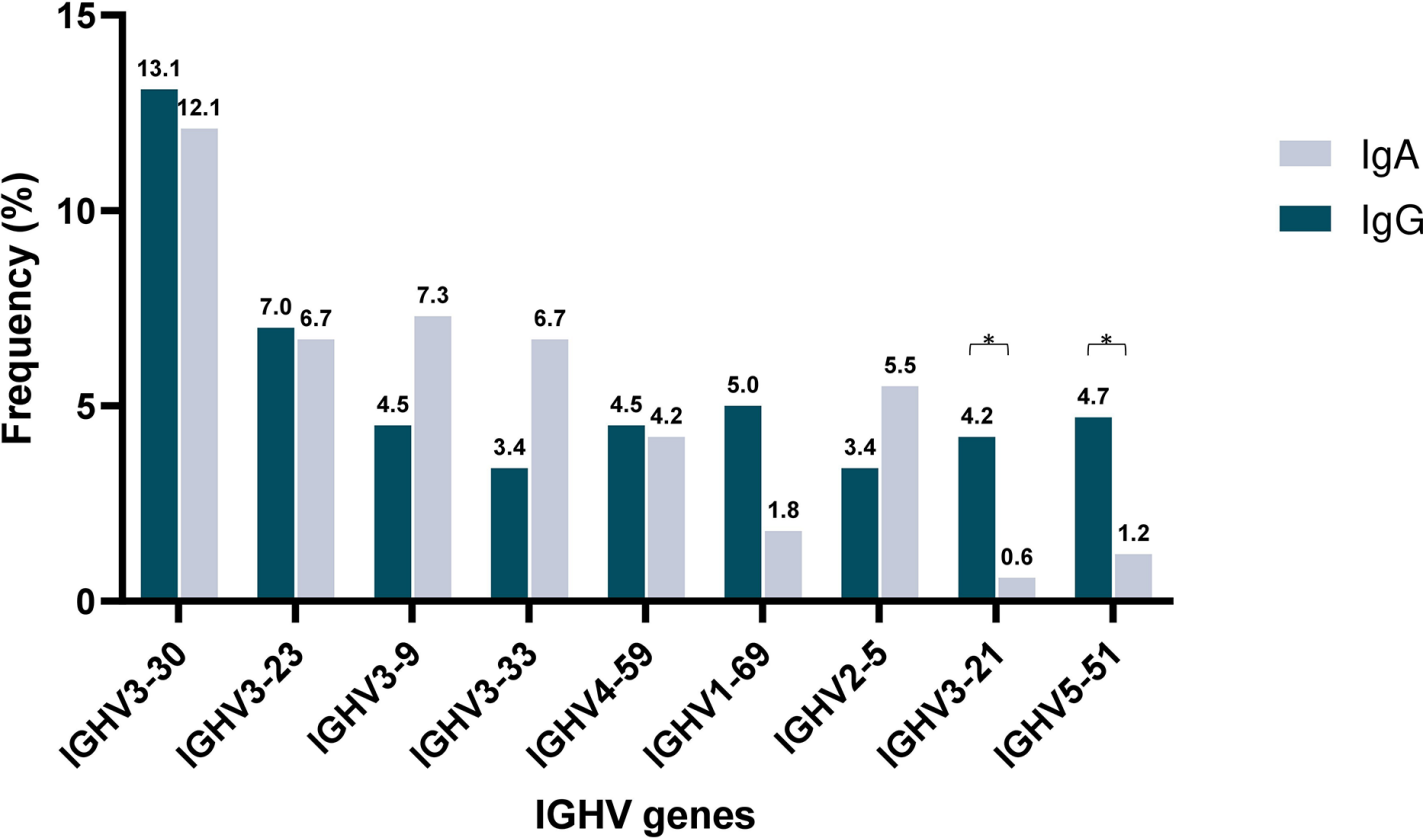
Shared and unique IGHV gene repertoires in IgA versus IgM multiple myeloma. Comparison of the most frequently used IGHV genes in IgA (blue) and IgG (gray) MM patients. Asterisks indicate the statistically significant differences between the two groups (p-value ≤ 0.05(*)).

Subsequently, IGHV gene repertoires were studied separately in IgG MM and IgA MM. Predominance of the IGHV3 subgroup was evident in both groups, accounting for 181/358 rearrangements (50.6%) in the IgG group and 98/165 rearrangements (59.4%) in the IgA group. Eight IGHV genes were characterized as frequent in the IgG group and 9 in the IgA group, respectively. Of these, only 4 IGHV genes, namely IGHV3-23, IGHV3-30, IGHV3-9 and IGHV4-59, were characterized as frequent in both groups. The remaining frequent IGHV genes showed variations in utilization frequency among the 2 MM types, with some differences reaching statistical significance. The most prominent differences concerned the IGHV3-21 gene, which exhibited a frequency of 4.2% in IgG MM versus only 0.6% in IgA MM (p-value <0.05), and the IGHV5-51 gene, accounting for 4.7% in IgG MM versus 1.2% in IgA MM (p-value=0.05) (**Supplemental Table 2**) (**Figure 1**).

### Shared and distinct VH CDR3 characteristics in IgA versus IgG multiple myeloma

#### IGHD gene repertoires

At cohort level, IGHD3 subgroup genes predominated (195/523 cases; 37.3%) followed by IGHD2 (99/523 cases; 18.3%) and IGHD6 genes (86/523 cases; 16.4%) (**Supplemental Table 3**). In total, 25 distinct IGHD genes were identified in the present cohort (**Supplemental Table 4**); the most frequent was IGHD3-10 (54/523 cases; 10.3%), followed by IGHD3-3 (45/523 cases; 8.6%) and IGHD3-22 (44/523 cases; 8.4%).

IGHD gene repertoire analysis was also performed in relation to heavy chain isotype expression, which confirmed the predominance of IGHD3 subgroup genes in both MM groups. However, the second most common IGHD gene subgroup differed, being IGHD2 in IgG MM versus IGHD6 in IgA MM; moreover, IGHD1 genes were twice as frequent in IgG versus IgA MM (p-value <0.05) (**Supplemental Table 3**). At the individual IGHD gene level, the only significant difference concerned the IGHD5-12 gene that was more frequent in IgA MM compared to IgG MM (6.7% versus 2.5%, respectively; p-value <0.05) (**Supplemental Table 4**) **(** **Supplemental Figure 1**).

Analysis of the IGHD gene reading frames (RF) revealed an overrepresentation of RF2 in both IgA MM and IgG MM (46.6% in both groups) (**Supplemental Table 5**). RF1 and RF3 displayed similar relative frequencies in IgA MM (45 cases; 27.2% and 43 cases; 26%, respectively). In contrast, in IgG MM RF2 was followed by RF3 (118 cases, 32.9%), while RF1 displayed the smallest relative frequency (73 cases, 20.3%).

Subsequently, the distribution of RFs was compared at the individual IGHD gene level. Focusing on IGHD genes that were frequent in both MM types, we noted that the utilization of the IGHD3-10 gene in RF2 was significantly more frequent in IgG MM compared to IgA MM (57.9% versus 33.3%, p-value <0.05), whereas RF3 predominated in cases expressing the IGHD5-12 gene belonging to IgA MM versus IgG MM (63.6% versus 57.1%, p-value <0.05) (**Supplemental Table 6**).

### IGHJ gene repertoires

The IGHJ4 gene was identified in 252/523 rearrangements of the total cohort (48.2%), followed by IGHJ6 (103/523 rearrangements; 19.7%) (**Supplemental Table 7**). At the heavy chain isotype level, the IGHJ4 gene was the most common in both IgG and IgA MM, followed in both cases by the IGHJ6 gene (**Supplemental Table 7**).

### Biased pairings of IGHV/IGHD/IGHJ genes

**Figure 2:**
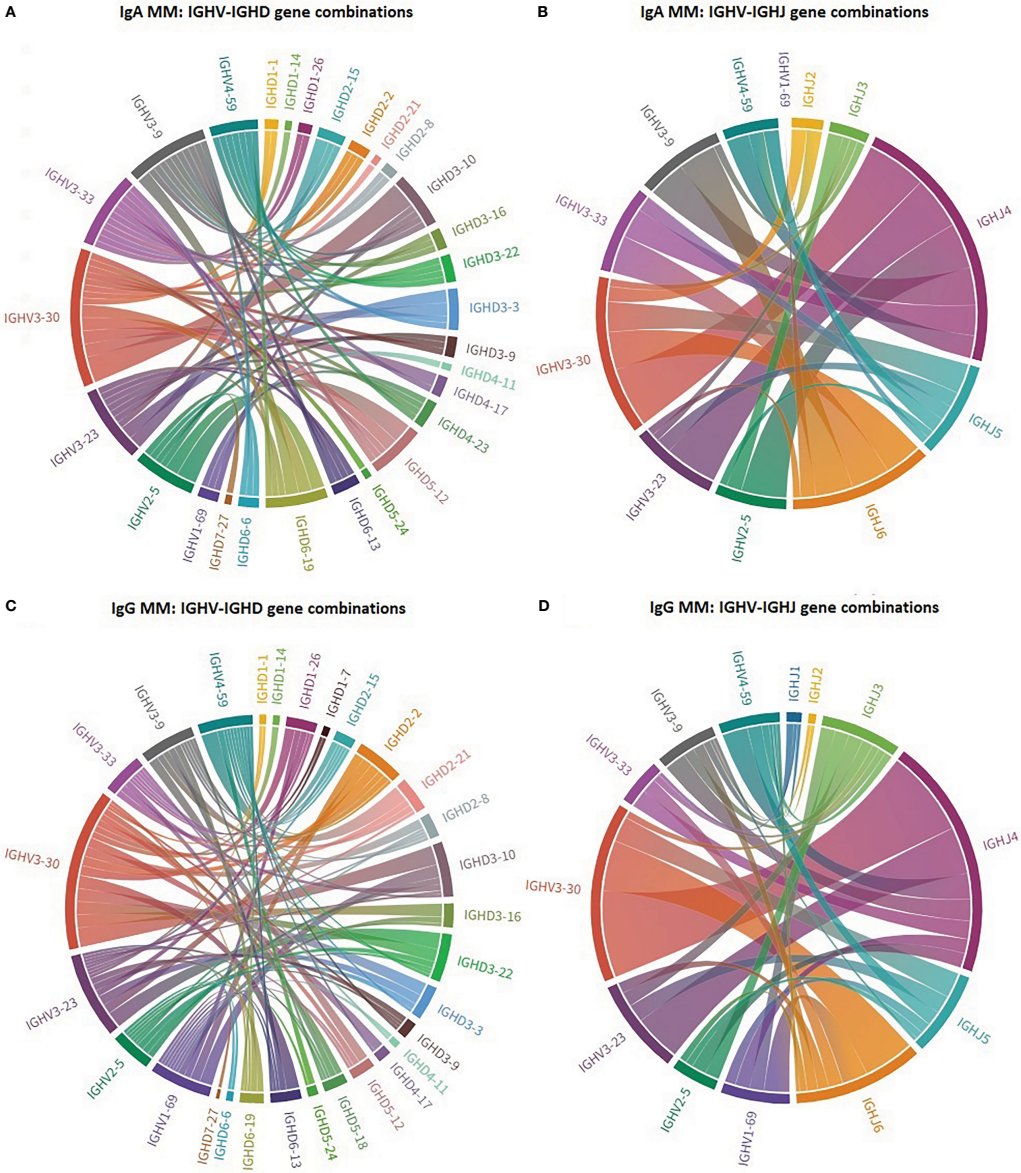
Biased IG gene pairings in multiple myeloma. Circos plot presenting IGHV-IGHD-IGHJ combinations of the 7 most frequent IGHV genes in IgA MM and IgG MM. The Circos software package was used to depict the predominant combinations between IGHV and IGHD genes [**(A)** IgA and **(C)** IgG patients] as well as between IGHV and IGHJ genes [**(B)** IgA and **(D)** IgG patients] in both MM groups.

Distinct patterns of associations of certain IGHV genes with IGHD and IGHJ genes were identified between IgA MM versus IgG MM (**Figure 2**). The most striking cases concerned: (i) IGHV3-30 in IgA MM, where 4/20 cases (20%) recombined with IGHD3-10, (ii) IGHV4-59 in IgG MM, where 5/16 cases (31%) recombined with IGHD2-21.

### VH CDR3 properties

**Figure 3:**
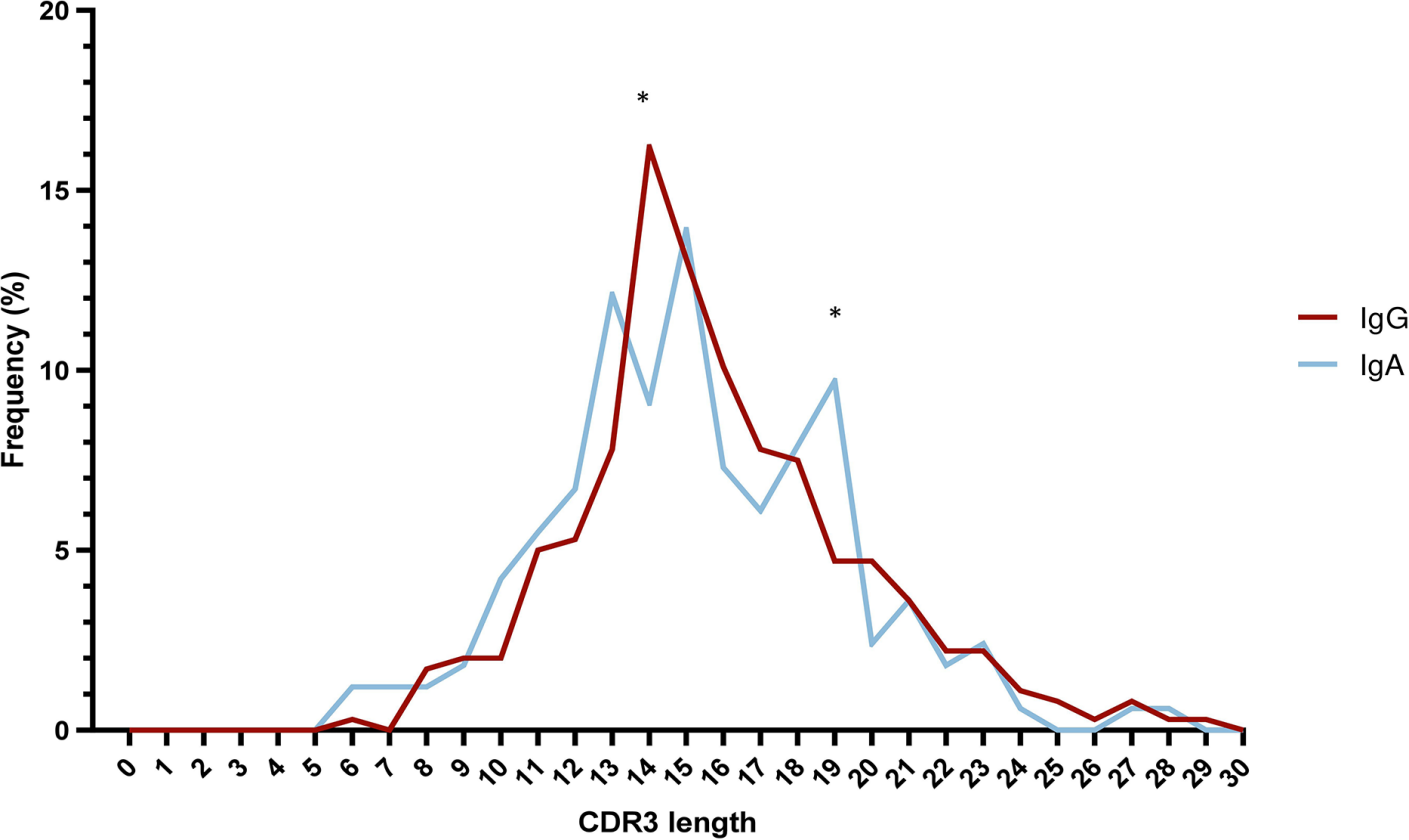
Skewed VH CDR3 length distribution in multiple myeloma. Distribution of VH CDR3 length of IgA MM (blue) and IgG MM (red). Significant differences between the two MM types are observed for VH CDR3 lengths of 14 aa and 19 aa. Asterisks indicate the statistically significant differences between the two groups (p-value ≤ 0.05 (*)).

The median VH CDR3 length at cohort level was 15 amino acids (aa) (range: 6-29), with no differences between IgA MM versus IgG MM. However, statistically significant differences were identified regarding the distribution of IgA and IgG cases based on the VH CDR3 length. More particularly, a higher frequency of IgG cases expressed 14 aa long VH CDR3 (58/358 IgG cases; 16.2%) compared to IgA cases (15/165 cases; 9.1%) (p-value <0.05). The opposite trend was observed for cases carrying VH CDR3 of 19 amino acids, which were enriched in the IgA group (16/165 cases; 9.7%) compared to the IgG group (17/358 cases; 4.7%) (p-value <0.05) (**Figure 3**) (**Supplemental Table 8**).

All productive IgA and IgG MM IGHV-IGHD-IGHJ gene rearrangements of the present cohort were subjected to analysis for VH CDR3 stereotypy using our established bioinformatics (25). However, this analysis did not identify clusters of immunogenetically related sequences, supporting that VH CDR3 stereotypy does not occur (or is very infrequent) in MM.

### Differential imprints of somatic hypermutation in subgroups of IgA versus IgG multiple myeloma

Cases were classified following the germline identity % (GI%) into the following subgroups: (i) truly unmutated [=100%], (ii) minimally mutated [>=99% and <100%], (iii) borderline mutated [>=97% and <99%], (iv) mutated [>=95% and <97%] and (v) heavily mutated [<95%]). The vast majority of rearrangements (471/523 cases, 90.1%) were heavily mutated (**Supplemental Table 9**), with a median GI of 92%; there was no difference in the median percent GI values burden between IgA versus IgG MM (91.3 GI% in IgA MM versus 92% in IgG MM). Only a single rearrangement from the IgA group was truly unmutated.

**Figure 4:**
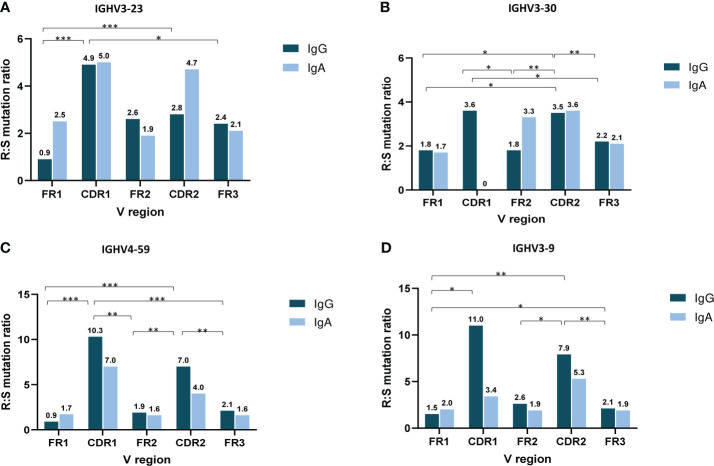
Topology of somatic hypermutation in IgA versus IgG multiple myeloma. Ratio of replacement to silent mutations (R:S) in the VH FRs and VH CDRs in cases expressing the 4 commonly frequent IGHV genes of MM patients with IgG and IgA isotypes: IGHV3-23 **(A)**, IGHV3-30 **(B)**, IGHV4-59 **(C)** and IGHV3-9 **(D)**. Asterisks indicate the statistically significant differences between the VH FR and VH CDR regions (p-value ≤ 0.05(*), p-value ≤ 0.01 (**) and p-value ≤ 0.001 (***)).

The analysis of SHM targeting at the individual IGHV gene level initially focused on IGHV genes that were characterized as frequent in both the IgG and IgA MM groups, namely IGHV3-23, IGHV3-30, IGHV3-9 and IGHV4-59. Cases expressing the IGHV3-23 and IGHV4-59 genes displayed significantly higher R:S ratios in VH CDRs versus VH FRs in IgG MM (p-value <0.001 and p-value < 0.01, respectively). When looking at individual regions of the VH domain, both the IgG and IgA groups displayed higher R:S ratios in VH CDR1 and VH CDR2 versus the VH FR regions (**Figure 4**). In contrast, significant differences were identified regarding the R:S ratios in the VH CDR1 domain between IgA and IgG patients carrying the IGHV1-18, IGHV3-48 and IGHV5-51 genes. In detail, the R:S ratio in the VH CDR1 of IGHV1-18 and IGHV5-51 gene rearrangements in patients with IgG MM was significantly higher compared to the respective IgA MM patients (10.5 in IgG versus 0.7 in IgA for IGHV1-18 rearrangements and 2.5 in IgG versus 0 in IgA for IGHV5-51 rearrangements, respectively; p-values <0.05 in both cases). The opposite trend was observed in cases expressing the IGHV3-48 gene, where the R:S ratio in the VH CDR1 in patients with IgG MM was significant lower compared to the respective ratio in IgA MM patients (0 in IgG versus 1.3 in IgA, respectively; p-value <0.05) (**Supplemental Table 10**).

Using the NetNglyc tool we detected N-glycosylation sequons in 93/523 IGHV-IGHD-IGHJ gene rearrangements (17.8%), with no significant differences between IgA MM (23/165 cases, 13.9%) versus IgG MM (70/358 cases, 19.5%) (p-value = 0.15). Of these, 38 cases carried novel N-glycosylation sequons introduced by SHM, again with no significant differences in frequency between IgA MM (8/23 cases, 34.8%) versus IgG MM (30/70 cases, 42.8%) (**Supplemental Table 11**).

Removal of germline encoded N-glycosylation sequons due to SHM was also observed in 18/430 cases (4.2%), again with a similar distribution among the 2 MM types [7/142 IgA cases (4.9%) versus 11/288 IgG cases (3.8%)]. Interestingly, 5/18 cases where such removal was documented expressed the IGHV4-39 gene; in 4/5 cases (2 each concerning IgA or IgG MM) this was due to a recurrent Proline for Lysine substitution at position VH FR3 69 (**Supplemental Table 12**).

## Discussion

In the present study we assessed the impact of antigen selection in shaping the IG gene repertoire of MM through an in-depth analysis of the clonotypic IGHV-IGHD-IGHJ gene rearrangements in patients expressing alpha or gamma isotypes.

Overall, the IGH gene repertoire in the present series was similar to those reported in previous reports (6, 7, 19), with the IGHV3-23, IGHV3-30, IGHV3-9 and IGHV4-59 genes predominating. However, certain IGHV or IGHD genes (e.g. IGHV2-5 and IGHD5-12) are herein reported as frequent for the first time, highlighting the added value of our study in offering a more comprehensive view of the immunogenetic landscape of MM. In keeping with the literature (19, 20), IGHV3 subgroup genes predominated in both IgA MM and IgG MM. Such predominance has also been noted in B cell lymphoproliferations, including monoclonal gammopathy of undetermined significance (MGUS) (27–29), chronic lymphocytic leukemia (CLL) and mantle cell lymphoma (MCL) (30, 31). A significant difference concerned IGHV4-34 that is conspicuously infrequent (<1%) in MM, yet it is very frequent in both hematologic malignancies, such as CLL and MCL (25, 31). Moreover, our cohort was notable for underrepresentation of the IGHV1-69, IGHV3-7 and IGHV1-8 genes, which have been reported as frequent in CLL and/or MCL (30, 31).

Studies in memory B cells have not identified differences in IG gene repertoires between IgA versus IgG memory B cells (10, 12, 32, 33). On these grounds, the herein reported distinctive features between IgA versus IgG MM do not appear to reflect the normal B cell differentiation, but rather allude to distinct selection forces shaping, at least in part, the respective BcR IG repertoires. This argument was reinforced by our finding of differential patterns of associations of certain IGHV genes with certain IGHD and IGHJ genes in IgG MM versus IgA MM types.

Overall, the present cohort of MM cases resembled normal B cells in terms of VH CDR3 length distribution and amino acid composition (34, 35), in sharp contrast to other malignancies of mature B cells e.g. chronic lymphocytic leukemia (CLL), mantle cell lymphoma (MCL) or marginal zone lymphoma (MZL) (31, 36, 37). In keeping with the literature (19, 20) and in contrast with CLL, MCL or splenic marginal zone lymphoma (SMZL) (31, 38–40), VH CDR3 stereotypy was not observed in our cohort. Of note, however, when considering IgG MM and IgA MM cases separately, skewing to particular CDR3 lengths was noted, in some instances reaching statistical significance. Considering the fundamental role of the VH CDR3 in antigen recognition, at least in the pre-immune repertoire (41), this observation supports differences at the level of selection of the corresponding MM progenitor cells, particularly regarding the type of selecting elements, the timing and the precise location of antigenic interactions within the secondary lymphoid tissues.

The vast majority of cases in our cohort bore a significant number of SHMs, in line with previous studies (6, 7, 42), supporting the notion that the MM transforming events concern post-germinal center B cell progenitors (8, 43). This finding, along with SHM profiles characteristic of encounter with classical antigen(s), typified by preferential targeting of replacement mutations in VH CDRs rather than VH FRs, further corroborates the notion that the MM progenitor cells were selected by antigens during their process of differentiation into plasma cells (9, 44). A noteworthy novel finding concerned the differences observed in at least some subgroups of IgA MM versus IgG MM cases expressing BcR IG with the same IGHV gene. On these grounds, we argue that not only antigen exposure trajectories but also affinity maturation processes may differ for IgA MM versus IgG MM, further underlining the role of microenvironmental interactions in the pathogenesis of the disease. This notion is further supported by a recent study on transgenic mice reporting the existence of similar SHM patterns between two B cell subpopulations located at different tissues, i.e. Peyer’s Patches germinal center B cells and splenic B cells (45). In this context, the identification of different SHM imprints in IgA compared to IgG MM plasma cells may allude to distinct modes of antigen exposure of MM progenitors in different microenvironmental niches.

N-glycosylation concerns the modification of IGs (or other proteins) by the addition of N-glycans to specific sequons; this modification can have a significant impact on the structure, stability, and biological function of the glycosylated molecules (46). Novel N-glycosylation sites introduced by SHM in the clonotypic IG are a very frequent feature in follicular lymphoma (FL) (~80%) and diffuse large B-cell lymphoma (DLCL) (51% in germinal-center B-cell–like DLBCL and 6% in activated B-cell–like DLBCL) (47–50). Furthermore, N-glycosylation of the clonotypic BcR IG has also been reported, albeit less frequently, in plasma cell disorders and B cell malignancies, such as AL amyloidosis (51–53). Prompted by evidence that SHM-induced changes in N-glycosylation of the IG variable domains is a mechanism redeeming B cells away from autoreactivity (54), here we sought to explore whether SHM within the clonotypic VH domain may lead to the creation of novel N-glycosylation sites or the disruption of germline-encoded ones. This is of potential clinical relevance if one also considers the generally low incidence of autoreactive phenomena in MM. Interestingly, examination of the particular SHMs leading to the creation and/or disruption of N-glycosylation sequons revealed the existence of recurrent amino acid substitutions at particular positions in certain IGHV genes (e.g. IGHV1-46 and IGHV4-39), albeit with no distinguishing features between IgA MM versus IgG MM.

In conclusion, in-depth analysis of the IG gene repertoire in the largest so far series of MM patients offers evidence supporting a role for antigens in disease pathogenesis. Distinct immunogenetic profiles in IgA MM versus IgG MM allude to distinct ontogenetic trajectories for particular subtypes of MM with potential impact on clonal behavior and clinical outcome.

## Data availability statement

The raw data supporting the conclusions of this article will be made available by the authors, without undue reservation.

## Ethics statement

The studies involving human participants were reviewed and approved by Centre for Research and Technology Hellas, Thessaloniki, Greece. The patients/participants provided their written informed consent to participate in this study.

## Author contributions

GG performed research, analyzed data and wrote the paper. AA supervised research and wrote the paper; GK performed statistical analysis. CL, AP, AM, EG performed research. KC, EH, MP, ET, CJ, IS, SF, ML, RS, CB provided samples and supervised research. KS designed the study, supervised research and wrote the paper. All authors contributed to the article and approved the submitted version.
