# Supplementary material for: Differences in the immunoglobulin gene repertoires of IgG versus IgA multiple myeloma allude to distinct immunopathogenetic trajectories
Source: Front Oncol. 2023 Feb 8;13:1123029. doi: 10.3389/fonc.2023.1123029 (PMC9945080; doi:10.3389/fonc.2023.1123029)
Supplement: Supplementary file 1 [file Presentation_1.pptx]

## Slide 1
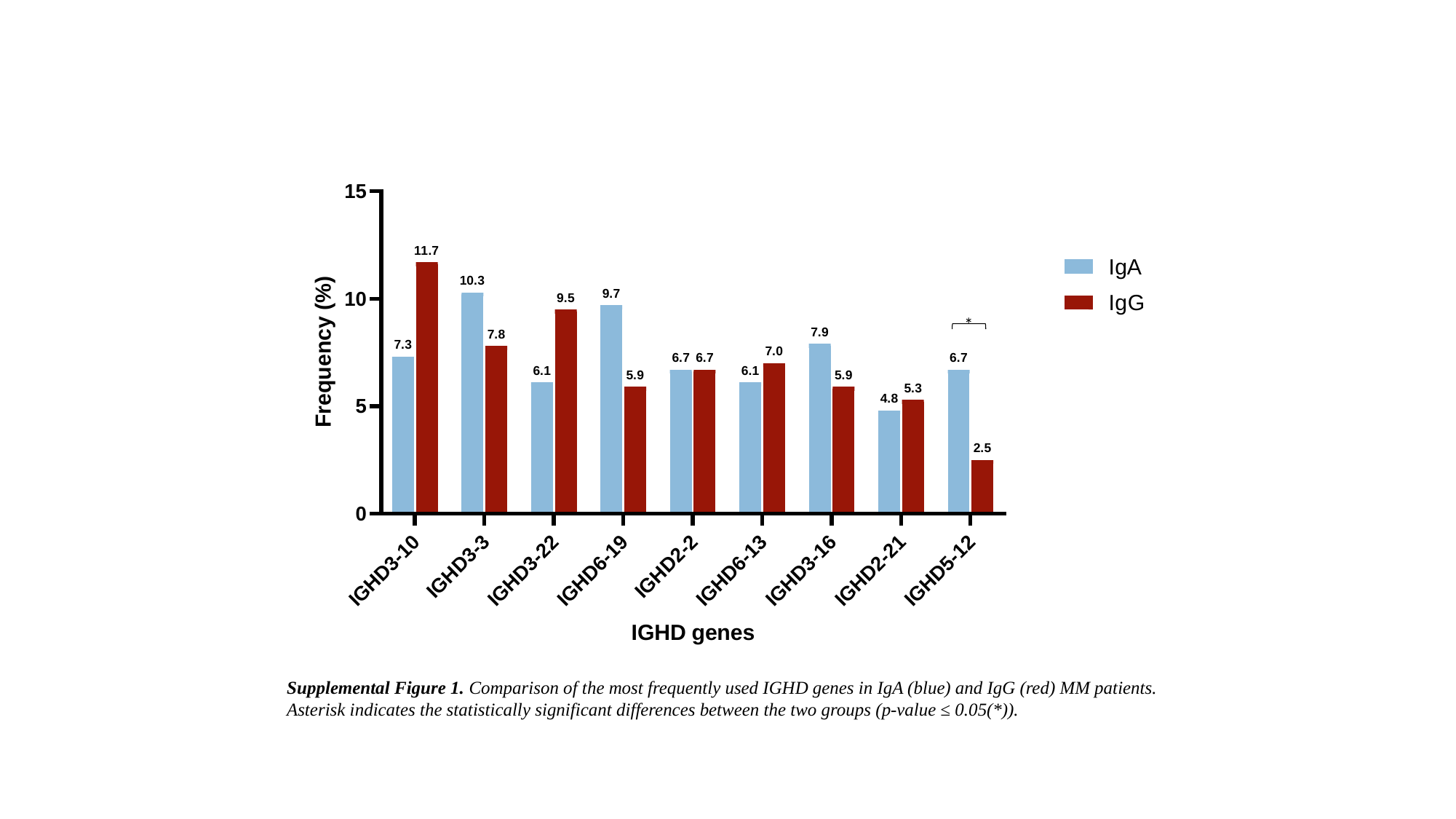

*
Supplemental Figure 1. Comparison of the most frequently used IGHD genes in IgA (blue) and IgG (red) MM patients. Asterisk indicates the statistically significant differences between the two groups (p-value ≤ 0.05(*)).
