# Supplementary material for: Differences in the immunoglobulin gene repertoires of IgG versus IgA multiple myeloma allude to distinct immunopathogenetic trajectories
Source: Front Oncol. 2023 Feb 8;13:1123029. doi: 10.3389/fonc.2023.1123029 (PMC9945080; doi:10.3389/fonc.2023.1123029)
Supplement: Supplementary file 2 [file Table_1.docx]

**Supplemental Material**

**Supplemental Tables**

**Supplemental Table 1.** Distribution of IGHV gene subgroups in the present series at cohort level as well as in the IgG and IgA MM groups.

|  | MM all | | | IgG MM | | | IgA MM | | |  | |
| --- | --- | --- | --- | --- | --- | --- | --- | --- | --- | --- | --- |
| IGHV gene subgroup | **No** | **%** | **No** | | **%** | **No** | | **%** | p-**value** | |  |
| IGHV1 | 66 | 12.9 | 50 | | 14.0 | 16 | | 9.7 | 0.13 | |  |
| IGHV2 | 45 | 8.6 | 33 | | 9.2 | 12 | | 7.3 | 0.62 | |  |
| IGHV3 | 279 | 53.3 | 181 | | 50.6 | 98 | | 59.4 | 0.06 | |  |
| IGHV4 | 96 | 18.4 | 65 | | 18.2 | 31 | | 18.8 | 0.90 | |  |
| IGHV5 | 31 | 5.9 | 23 | | 6.4 | 8 | | 4.8 | 0.69 | |  |
| IGHV6 | 5 | 1.0 | 5 | | 1.4 | 0 | | 0.0 | 0.33 | |  |
| IGHV7 | 1 | 0.2 | 1 | | 0.3 | 0 | | 0.0 | 1.00 | |  |

**Supplemental Table 2.** IGHV gene repertoire in the present series at cohort level as well as in the IgG and IgA MM groups.

|  | MM all | | IgG MM | | IgA MM | |  |
| --- | --- | --- | --- | --- | --- | --- | --- |
| IGHV gene | **No** | **%** | **No** | **%** | **No** | **%** | **p-value** |
| IGHV1-18 | 14 | 2.7 | 8 | 2.2 | 6 | 3.6 | 0.39 |
| IGHV1-2 | 12 | 2.3 | 10 | 2.8 | 2 | 1.2 | 0.36 |
| IGHV1-24 | 9 | 1.7 | 6 | 1.7 | 3 | 1.8 | 1.00 |
| IGHV1-3 | 2 | 0.4 | 1 | 0.3 | 1 | 0.6 | 0.53 |
| IGHV1-46 | 6 | 1.1 | 5 | 1.4 | 1 | 0.6 | 0.67 |
| IGHV1-58 | 1 | 0.2 | 1 | 0.3 | 0 | 0.0 | 1.00 |
| IGHV1-69 | 21 | 4.0 | 18 | 5.0 | 3 | 1.8 | 0.10 |
| IGHV1-69-2 | 1 | 0.2 | 0 | 0.0 | 1 | 0.6 | 0.32 |
| IGHV1-8 | 2 | 0.4 | 1 | 0.3 | 1 | 0.6 | 0.53 |
| IGHV2-26 | 11 | 2.1 | 10 | 2.8 | 1 | 0.6 | 0.19 |
| IGHV2-5 | 21 | 4.0 | 12 | 3.4 | 9 | 5.5 | 0.34 |
| IGHV2-70 | 13 | 2.5 | 11 | 3.1 | 2 | 1.2 | 0.24 |
| IGHV3-11 | 15 | 2.9 | 9 | 2.5 | 6 | 3.6 | 0.57 |
| IGHV3-13 | 1 | 0.2 | 0 | 0.0 | 1 | 0.6 | 0.32 |
| IGHV3-15 | 16 | 3.0 | 9 | 2.5 | 7 | 4.2 | 0.29 |
| IGHV3-20 | 3 | 0.6 | 2 | 0.6 | 1 | 0.6 | 1.00 |
| IGHV3-21 | 16 | 3.0 | 15 | 4.2 | 1 | 0.6 | **0.03** |
| IGHV3-23 | 36 | 6.9 | 25 | 7.0 | 11 | 6.7 | 1.00 |
| IGHV3-30 | 67 | 12.8 | 47 | 13.1 | 20 | 12.1 | 0.78 |
| IGHV3-30-3 | 14 | 2.7 | 10 | 2.8 | 4 | 2.4 | 1.00 |
| IGHV3-33 | 23 | 4.4 | 12 | 3.4 | 11 | 6.7 | 0.11 |
| IGHV3-43 | 7 | 1.3 | 4 | 1.1 | 3 | 1.8 | 0.68 |
| IGHV3-48 | 8 | 1.5 | 6 | 1.7 | 2 | 1.2 | 1.00 |
| IGHV3-49 | 3 | 0.6 | 3 | 0.8 | 0 | 0.0 | 0.56 |
| IGHV3-53 | 4 | 0.8 | 2 | 0.6 | 2 | 1.2 | 0.59 |
| IGHV3-64 | 1 | 0.2 | 1 | 0.3 | 0 | 0.0 | 1.00 |
| IGHV3-64D | 4 | 0.8 | 4 | 1.1 | 0 | 0.0 | 0.31 |
| IGHV3-66 | 2 | 0.4 | 1 | 0.3 | 1 | 0.6 | 0.53 |
| IGHV3-69-1 | 1 | 0.2 | 1 | 0.3 | 0 | 0.0 | 1.00 |
| IGHV3-7 | 16 | 3.0 | 8 | 2.2 | 8 | 4.8 | 0.17 |
| IGHV3-74 | 13 | 2.5 | 6 | 1.7 | 7 | 4.2 | 0.13 |
| IGHV3-73 | 1 | 0.2 | 0 | 0.0 | 1 | 0.6 | 0.32 |
| IGHV3-9 | 28 | 5.4 | 16 | 4.5 | 12 | 7.3 | 0.21 |
| IGHV4-28 | 1 | 0.2 | 1 | 0.3 | 0 | 0.0 | 1.00 |
| IGHV4-30-2 | 1 | 0.2 | 0 | 0.0 | 1 | 0.6 | 0.32 |
| IGHV4-30-4 | 9 | 1.7 | 7 | 2.0 | 2 | 1.2 | 0.73 |
| IGHV4-31 | 8 | 1.5 | 6 | 1.7 | 2 | 1.2 | 1.00 |
| IGHV4-34 | 4 | 0.8 | 2 | 0.6 | 2 | 1.2 | 0.59 |
| IGHV4-38-2 | 2 | 0.4 | 1 | 0.3 | 1 | 0.6 | 0.53 |
| IGHV4-39 | 18 | 3.4 | 15 | 4.2 | 3 | 1.8 | 0.20 |
| IGHV4-4 | 17 | 3.2 | 11 | 3.1 | 6 | 1.7 | 0.79 |
| IGHV4-59 | 23 | 4.4 | 16 | 4.5 | 7 | 4.2 | 1.00 |
| IGHV4-61 | 11 | 2.1 | 6 | 1.7 | 5 | 3.0 | 0.34 |
| IGHV5-10-1 | 12 | 2.3 | 6 | 1.7 | 6 | 3.6 | 0.21 |
| IGHV5-51 | 19 | 3.6 | 17 | 4.7 | 2 | 1.2 | **0.05** |
| IGHV6-1 | 5 | 0.9 | 5 | 1.4 | 0 | 0.0 | 0.33 |
| IGHV7-4 | 1 | 0.2 | 1 | 0.3 | 0 | 0.0 | 1.00 |

**Supplemental Table 3.** Distribution of IGHD gene subgroups in the present series at cohort level as well as in the IgG and IgA MM groups.

|  | MM all | | | IgG MM | | | IgA MM | | |  | |
| --- | --- | --- | --- | --- | --- | --- | --- | --- | --- | --- | --- |
| IGHD gene subgroup | **No** | **%** | **No** | | **%** | **No** | | **%** | **p-value** | |  |
| IGHD1 | 54 | 10.3 | 44 | | 12.3 | 10 | | 6.1 | 0.03 | |  |
| IGHD2 | 99 | 18.3 | 68 | | 19.0 | 31 | | 18.8 | 1.00 | |  |
| IGHD3 | 195 | 37.3 | 138 | | 38.5 | 57 | | 34.5 | 0.44 | |  |
| IGHD4 | 36 | 6.9 | 23 | | 6.4 | 13 | | 7.9 | 0.58 | |  |
| IGHD5 | 47 | 9.0 | 29 | | 8.1 | 18 | | 10.9 | 0.32 | |  |
| IGHD6 | 86 | 16.4 | 54 | | 15.1 | 32 | | 19.4 | 0.25 | |  |
| IGHD7 | 6 | 1.1 | 2 | | 0.6 | 4 | | 2.4 | 0.08 | |  |

**Supplemental Table 4.** IGHD gene repertoire in the present series at cohort level as well as in the IgG and IgA MM groups.

|  | MM all | | IgG MM | | IgA MM | |  |
| --- | --- | --- | --- | --- | --- | --- | --- |
| IGHD gene | **No** | **%** | **No** | **%** | **No** | **%** | **p-value** |
| IGHD1-1 | 9 | 1.7 | 6 | 1.7 | 3 | 1.8 | 1.00 |
| IGHD1-14 | 5 | 1.0 | 4 | 1.1 | 1 | 0.6 | 1.00 |
| IGHD1-20 | 5 | 1.0 | 5 | 1.4 | 0 | 0.0 | 0.33 |
| IGHD1-7 | 9 | 1.7 | 8 | 2.2 | 1 | 0.6 | 0.28 |
| IGHD2-15 | 24 | 4.6 | 16 | 4.5 | 8 | 4.9 | 0.83 |
| IGHD1-26 | 26 | 5.0 | 21 | 5.9 | 5 | 3.0 | 0.20 |
| IGHD2-2 | 35 | 6.7 | 24 | 6.7 | 11 | 6.7 | 1.00 |
| IGHD2-21 | 27 | 5.2 | 19 | 5.3 | 8 | 4.8 | 1.00 |
| IGHD2-8 | 13 | 2.5 | 9 | 2.5 | 4 | 2.4 | 1.00 |
| IGHD3-10 | 54 | 10.3 | 42 | 11.7 | 12 | 7.3 | 0.13 |
| IGHD3-16 | 34 | 6.5 | 21 | 5.9 | 13 | 7.9 | 0.45 |
| IGHD3-22 | 44 | 8.4 | 34 | 9.5 | 10 | 6.1 | 0.24 |
| IGHD3-3 | 45 | 8.6 | 28 | 7.8 | 17 | 10.3 | 0.40 |
| IGHD3-9 | 18 | 3.4 | 13 | 3.6 | 5 | 3.0 | 0.80 |
| IGHD4-11 | 8 | 1.5 | 4 | 1.1 | 4 | 2.4 | 0.27 |
| IGHD4-17 | 18 | 3.4 | 13 | 3.6 | 5 | 3.0 | 0.80 |
| IGHD4-23 | 10 | 1.9 | 6 | 1.7 | 4 | 2.4 | 0.52 |
| IGHD5-12 | 20 | 3.8 | 9 | 2.5 | 11 | 6.7 | **0.03** |
| IGHD5-18 | 16 | 3.1 | 13 | 3.6 | 3 | 1.8 | 0.41 |
| IGHD5-24 | 11 | 2.1 | 7 | 2.0 | 4 | 2.4 | 0.75 |
| IGHD6-6 | 13 | 2.5 | 8 | 2.2 | 5 | 3.0 | 0.56 |
| IGHD6-13 | 35 | 6.7 | 25 | 7.0 | 10 | 6.1 | 0.85 |
| IGHD6-19 | 37 | 7.1 | 21 | 5.9 | 16 | 9.7 | 0.14 |
| IGHD6-25 | 1 | 0.2 | 0 | 0.0 | 1 | 0.6 | 0.32 |
| IGHD7-27 | 6 | 1.1 | 2 | 0.6 | 4 | 2.4 | 0.08 |

**Supplemental Table 5.** Distribution of IGHD gene reading frames (RFs) in different MM groups of the present cohort.

|  | MM all | | IgG MM | | IgA MM | |  |
| --- | --- | --- | --- | --- | --- | --- | --- |
| IGHD gene Reading Frame | **No** | **%** | **No** | **%** | **No** | **%** | **p-value** |
| RF1 | 118 | 22.5 | 73 | 20.3 | 45 | 27.2 | 0.10 |
| RF2 | 244 | 46.6 | 167 | 46.6 | 77 | 46.6 | 1.00 |
| RF3 | 161 | 30.8 | 118 | 32.9 | 43 | 26 | 0.14 |

**Supplemental Table 6.** Distribution of IGHD gene reading frames (RFs) at the individual IGHD gene level in different MM groups of the present cohort.

|  | IgG MM | | | | | | | IgA MM | | | | | | |  | | |
| --- | --- | --- | --- | --- | --- | --- | --- | --- | --- | --- | --- | --- | --- | --- | --- | --- | --- |
|  | **RF1** | | **RF2** | | **RF3** | | **RF1** | | | **RF2** | | **RF3** | | **RF1** | | **RF2** | **RF3** |
| IGHD gene | **No** | **%** | **No** | **%** | **No** | **%** | **No** | | **%** | **No** | **%** | **No** | **%** | **p-value** | | | |
| IGHD1-1 | 2 | 33.3 | 1 | 16.7 | 3 | 50.0 | 1 | | 50.0 | 0 | 0.0 | 1 | 50.0 | 1.00 | | 1.00 | 1.00 |
| IGHD1-14 | 1 | 25.0 | 3 | 75.0 | 0 | 0.0 | 1 | | 100 | 0 | 0.0 | 0 | 0.0 | 1.00 | | 0.60 | - |
| IGHD1-20 | 2 | 50.0 | 0 | 0.0 | 2 | 50.0 | 0 | | 0.0 | 0 | 0.0 | 0 | 0.0 | - | | - | - |
| IGHD1-26 | 5 | 25.0 | 6 | 30.0 | 9 | 45.0 | 0 | | 0.0 | 3 | 75.0 | 1 | 25.0 | 0.18 | | 1.00 | 0.34 |
| IGHD1-7 | 2 | 33.3 | 2 | 33.3 | 2 | 33.3 | 1 | | 100 | 0 | 0.0 | 0 | 0.0 | 1.00 | | 0.86 | 0.93 |
| IGHD2-15 | 0 | 0.0 | 6 | 46.2 | 7 | 53.8 | 0 | | 0.0 | 7 | 87.5 | 1 | 12.5 | - | | 0.12 | 0.55 |
| IGHD2-2 | 3 | 14.3 | 8 | 38.1 | 10 | 47.6 | 1 | | 12.5 | 5 | 62.5 | 2 | 25.0 | 0.97 | | 0.75 | 0.57 |
| IGHD2-21 | 3 | 18.8 | 7 | 43.7 | 6 | 37.5 | 0 | | 0.0 | 3 | 42.9 | 4 | 57.1 | 0.43 | | 1.00 | 0.61 |
| IGHD2-8 | 1 | 14.2 | 3 | 42.9 | 3 | 42.9 | 2 | | 100 | 0 | 0.0 | 0 | 0.0 | 0.68 | | 0.60 | 0.66 |
| IGHD3-10 | 5 | 13.2 | 22 | 57.9 | 11 | 28.9 | 5 | | 55.6 | 3 | 33.3 | 1 | 11.1 | 0.66 | | **0.05** | 0.21 |
| IGHD3-16 | 2 | 10.5 | 13 | 68.4 | 4 | 21.1 | 2 | | 20.0 | 5 | 50.0 | 3 | 30.0 | 1.00 | | 0.99 | 0.64 |
| IGHD3-22 | 1 | 3.8 | 23 | 88.5 | 2 | 7.7 | 0 | | 0.0 | 8 | 88.9 | 1 | 11.1 | 1.00 | | 0.67 | 1.00 |
| IGHD3-3 | 2 | 7.7 | 16 | 61.5 | 8 | 30.8 | 2 | | 12.5 | 9 | 56.2 | 5 | 31.3 | 1.00 | | 0.70 | 0.57 |
| IGHD3-9 | 3 | 25.0 | 7 | 58.3 | 2 | 16.7 | 1 | | 25.0 | 3 | 75.0 | 0 | 0.0 | 0.97 | | 1.00 | 0.93 |
| IGHD4-11 | 0 | 0.0 | 2 | 66.7 | 1 | 33.3 | 1 | | 25.0 | 3 | 75.0 | 0 | 0.0 | 0.81 | | 0.34 | 1.00 |
| IGHD4-17 | 0 | 0.0 | 6 | 54.5 | 5 | 45.5 | 0 | | 0.0 | 2 | 100 | 0 | 0.0 | - | | 1.00 | 0.36 |
| IGHD4-23 | 0 | 0.0 | 4 | 66.7 | 2 | 33.3 | 0 | | 0.0 | 1 | 25.0 | 3 | 75.0 | - | | 0.97 | 0.26 |
| IGHD5-12 | 2 | 28.6 | 1 | 14.3 | 4 | 57.1 | 3 | | 27.3 | 1 | 9.1 | 7 | 63.6 | 0.59 | | 1.00 | **0.02** |
| IGHD5-18 | 1 | 10.0 | 1 | 10.0 | 8 | 80.0 | 0 | | 0.0 | 2 | 66.7 | 1 | 33.3 | 1.00 | | 0.47 | 0.44 |
| IGHD5-24 | 1 | 20.0 | 0 | 0.0 | 4 | 80.0 | 0 | | 0.0 | 0 | 0.0 | 4 | 100 | 1.00 | |  | 0.30 |
| IGHD6-19 | 10 | 58.8 | 7 | 41.2 | 0 | 0.0 | 9 | | 64.3 | 4 | 28.6 | 1 | 7.1 | 0.54 | | 0.93 | 0.62 |
| IGHD6-13 | 7 | 33.3 | 9 | 42.9 | 5 | 23.8 | 4 | | 40.0 | 5 | 50.0 | 1 | 10.0 | 1.00 | | 0.90 | 0.87 |
| IGHD6-25 | 0 | 0.0 | 0 | 0.0 | 0 | 0.0 | 1 | | 100 | 0 | 0.0 | 0 | 0.0 | - | | - | - |
| IGHD6-6 | 4 | 66.7 | 2 | 33.3 | 0 | 0.0 | 2 | | 50.0 | 1 | 25.0 | 1 | 25.0 | 1.00 | | 1.00 | 0.62 |
| IGHD7-27 | 2 | 100 | 0 | 0.0 | 0 | 0 | 1 | | 33.3 | 1 | 33.3 | 1 | 33.3 | 1.00 | | 0.67 | 0.62 |

**Supplemental Table 7.** IGHJ gene repertoire in different patient groups of the present series.

|  | MM all | | IgG MM | | IgA MM | |  |
| --- | --- | --- | --- | --- | --- | --- | --- |
| IGHJ gene | **No** | **%** | **No** | **%** | **No** | **%** | **p-value** |
| IGHJ1 | 14 | 2.7 | 12 | 3.4 | 2 | 1.2 | 0.24 |
| IGHJ2 | 14 | 2.7 | 8 | 2.2 | 6 | 3.6 | 0.39 |
| IGHJ3 | 71 | 13.6 | 51 | 14.2 | 20 | 12.1 | 0.58 |
| IGHJ4 | 252 | 48.2 | 173 | 48.3 | 79 | 47.9 | 1.00 |
| IGHJ5 | 69 | 13.2 | 47 | 13.1 | 22 | 13.3 | 1.00 |
| IGHJ6 | 103 | 19.7 | 67 | 18.7 | 36 | 21.8 | 0.41 |

**Supplemental Table 8.** Distribution of the VH CDR3 length in different groups of MM patients.

|  | IgG MM | | | IgA MM | | |  | |
| --- | --- | --- | --- | --- | --- | --- | --- | --- |
| VH CDR3 length (aa) | **No** | **%** | **No** | | **%** | **p-value** | |  |
| 6 | 1 | 0.3 | 2 | | 1.2 | 0.24 | |  |
| 7 | 0 | 0.0 | 2 | | 1.2 | 0.10 | |  |
| 8 | 6 | 1.7 | 2 | | 1.2 | 1.00 | |  |
| 9 | 7 | 2.0 | 3 | | 1.8 | 1.00 | |  |
| 10 | 7 | 2.0 | 7 | | 1.2 | 0.15 | |  |
| 11 | 18 | 5.0 | 9 | | 5.5 | 0.83 | |  |
| 12 | 19 | 5.3 | 11 | | 6.7 | 0.55 | |  |
| 13 | 28 | 7.8 | 20 | | 12.1 | 0.14 | |  |
| 14 | 58 | 16.2 | 15 | | 9.1 | **0.03** | |  |
| 15 | 47 | 13.1 | 23 | | 13.9 | 0.78 | |  |
| 16 | 36 | 10.1 | 12 | | 7.3 | 0.33 | |  |
| 17 | 28 | 7.8 | 10 | | 6.1 | 0.59 | |  |
| 18 | 27 | 7.5 | 13 | | 7.9 | 0.86 | |  |
| 19 | 17 | 4.7 | 16 | | 9.7 | **0.03** | |  |
| 20 | 17 | 4.7 | 4 | | 2.4 | 0.24 | |  |
| 21 | 13 | 3.6 | 6 | | 3.6 | 1.00 | |  |
| 22 | 8 | 2.2 | 3 | | 1.8 | 1.00 | |  |
| 23 | 8 | 2.2 | 4 | | 2.4 | 1.00 | |  |
| 24 | 4 | 1.1 | 1 | | 0.6 | 1.00 | |  |
| 25 | 3 | 0.8 | 0 | | 0.0 | 0.56 | |  |
| 26 | 1 | 0.3 | 0 | | 0.0 | 1.00 | |  |
| 27 | 3 | 0.8 | 1 | | 0.6 | 1.00 | |  |
| 28 | 1 | 0.3 | 1 | | 0.6 | 0.53 | |  |
| 29 | 1 | 0.3 | 0 | | 0.0 | 1.00 | |  |

**Supplemental Table 9.** Distribution of the somatic hypermutation (SHM) status in different groups of MM patients.

|  | MM all | | IgG MM | | IgA MM | |  |
| --- | --- | --- | --- | --- | --- | --- | --- |
| Mutational status | **No** | **%** | **No** | **%** | **No** | **%** | **p-value** |
| truly unmutated [=100] | 1 | 0.2 | 0 | 0.0 | 1 | 0.6 | 0.69 |
| minimally mutated [>=99 and <100] | 0 | 0.0 | 0 | 0.0 | 0 | 0.0 | - |
| borderline mutated [>=97 and <99] | 3 | 0.6 | 9 | 2.5 | 0 | 0.0 | 0.58 |
| mutated [>=95 and <97] | 48 | 9.2 | 36 | 10.1 | 14 | 8.5 | 0.24 |
| heavily mutated [<95] | 471 | 90.1 | 313 | 87.4 | 150 | 90.9 | 0.22 |

**Supplemental Table 10.** Ratio of replacement to silent mutations (R:S) in VH FRs and VH CDRs of IGHV genes of MM patients belonging to the IgG (A) and IgA (B) groups.

|  | IgG MM | | | | | IgA MM | | | | | p-value | | | | |
| --- | --- | --- | --- | --- | --- | --- | --- | --- | --- | --- | --- | --- | --- | --- | --- |
| IGHV gene | **FR1** | **CDR1** | **FR2** | **CDR2** | **FR3** | **FR1** | **CDR1** | **FR2** | **CDR2** | **FR3** | **FR1** | **FR2** | **FR3** | **CDR1** | **CDR2** |
| IGHV1-18 | 2.3 | 10.5 | 0.8 | 2.4 | 2.4 | 0.5 | 0.7 | 2.5 | 8 | 1.9 | 0.33 | 0.12 | 0.66 | **0.04** | 0.28 |
| IGHV1-2 | 1.8 | 2.4 | 0.8 | 3.1 | 2.2 | 0 | 2 | 7 | 2 | 4.8 | 0.43 | 0.07 | **0.01** | 1.00 | 1.00 |
| IGHV1-24 | 2 | 8 | 2.7 | 3.3 | 2.6 | 0.8 | 0 | 0 | 7 | 4.3 | 1.00 | 1.00 | 0.40 | 1.00 | 0.87 |
| IGHV1-3 | 0 | 0 | 1 | 1 | 4 | 0 | 5 | 0 | 0 | 5.7 |  | 0.82 | 1.00 | - | 0.54 |
| IGHV1-46 | 0.6 | 3.5 | 2.3 | 7 | 2.1 | 0 | 5 | 1.5 | 3 | 2 | 0.86 | 1.00 | 1.00 | 1.00 | 1.00 |
| IGHV1-58 | 0 | 3 | 0.7 | 2 | 14 | 0 | 0 | 0 | 0 | 0 | - | - | - | - | - |
| IGHV1-69 | 1.1 | 5.9 | 1.1 | 2.6 | 1.8 | 0 | 2 | 2 | 10 | 2.5 | 0.32 | 1.00 | 0.42 | 0.46 | 0.34 |
| IGHV1-69-2 | 0 | 0 | 0 | 0 | 0 | 6 | 0 | 0 | 0.5 | 9 | - | - | - | - | - |
| IGHV1-8 | 3 | 0 | 0.3 | 3 | 1.8 | 0 | 0 | 0 | 1 | 9 | - | 1.00 | 0.37 | - | 1.00 |
| IGHV2-26 | 1 | 2.8 | 1.5 | 15 | 1.6 | 0 | 0 | 0 | 0 | 2 | - | - | 1.00 | 0.78 | 1.00 |
| IGHV2-5 | 0.9 | 2.8 | 1.2 | 11 | 2.1 | 1 | 6.3 | 2.8 | 5 | 1.7 | 1.00 | 0.35 | 0.13 | 0.31 | 0.85 |
| IGHV2-70 | 1.7 | 26 | 1 | 5 | 1.4 | 0 | 4 | 2 | 3 | 2 | 1.00 | 1.00 | 0.75 | 0.71 | 1.00 |
| IGHV3-11 | 2.2 | 8 | 1.6 | 8.8 | 1.4 | 1 | 1 | 2.2 | 3.1 | 2 | 0.63 | 0.82 | 0.31 | 0.37 | 0.09 |
| IGHV3-13 | 0 | 0 | 0 | 0 | 0 | 0 | 0 | 0 | 0 | 0 | - | - | - | - | - |
| IGHV3-15 | 1.3 | 0 | 13 | 3.2 | 1.6 | 0.6 | 8 | 1.5 | 2 | 2.1 | 0.56 | 0.10 | 0.59 | 0.92 | 0.78 |
| IGHV3-20 | 0 | 0 | 1 | 3 | 2 | 2 | 0 | 0 | 3 | 0.4 | 1.00 | 1.00 | 1.00 | - | 1.00 |
| IGHV3-21 | 4 | 2.9 | 1.3 | 3.9 | 1.2 | 3 | 0 | 3 | 1 | 1.5 | 1.00 | 0.87 | 1.00 | 1.00 | 0.89 |
| IGHV3-23 | 0.9 | 4.9 | 2.6 | 2.8 | 2.4 | 2.5 | 5 | 1.9 | 4.7 | 2.1 | 0.17 | 0.65 | 0.80 | 1.00 | 0.24 |
| IGHV3-30 | 1.8 | 3.6 | 1.8 | 3.5 | 2.2 | 1.7 | 0 | 3.3 | 3.6 | 2.1 | 1.00 | 0.12 | 0.74 | 0.86 | 0.76 |
| IGHV3-30-3 | 0 | 3 | 3.5 | 5.1 | 2.5 | 0 | 8 | 1.6 | 4.3 | 3.1 | - | 0.48 | 0.79 | 0.66 | 1.00 |
| IGHV3-33 | 2.7 | 2 | 3.6 | 4.7 | 1.2 | 1.2 | 6.5 | 1.5 | 5.6 | 2.3 | 0.60 | 0.29 | **0.05** | 0.20 | 1.00 |
| IGHV3-43 | 1.7 | 1.5 | 1.5 | 0 | 1.6 | 3 | 0 | 2 | 3.3 | 2.7 | 1.00 | 1.00 | 0.61 | 0.30 | 0.31 |
| IGHV3-48 | 1.5 | 0 | 1.5 | 3.9 | 1.5 | 2 | 1.3 | 0 | 0 | 2.7 | 1.00 | 0.42 | 0.86 | **0.02** | 0.37 |
| IGHV3-49 | 1.8 | 2 | 1.3 | 3.7 | 1.5 | 0 | 0 | 0 | 0 | 0 | - | - | - | - | - |
| IGHV3-53 | 0 | 0 | 7 | 4 | 1.4 | 0 | 6 | 0.5 | 4 | 2.6 | - | 0.08 | 0.57 | 0.54 | 1.00 |
| IGHV3-64 | 0 | 0 | 0 | 2 | 4 | 0 | 0 | 0 | 0 | 0 | - | - | - | - | - |
| IGHV3-64D | 0.5 | 6 | 1.3 | 4.5 | 1.5 | 0 | 0 | 0 | 0 | 0 | - | - | - | - | - |
| IGHV3-66 | 0 | 0 | 1 | 0.5 | 1.2 | 3 | 0 | 0 | 1 | 0 | 1.00 | 0.76 | 0.30 | - | 1.00 |
| IGHV3-69-1 | 0 | 0 | 0 | 6 | 0.4 | 0 | 0 | 0 | 0 | 0 | - | - | - | - | - |
| IGHV3-7 | 5.3 | 6 | 2.3 | 2.2 | 2.9 | 1.6 | 2.3 | 3.2 | 4 | 2.5 | 0.18 | 0.92 | 0.88 | 0.34 | 0.51 |
| IGHV3-73 | 0 | 0 | 0 | 0 | 0 | 0 | 0 | 0.3 | 5 | 1.6 | - | - | - | - | - |
| IGHV3-74 | 1.5 | 5.5 | 0.6 | 12 | 2.3 | 0.8 | 0 | 7 | 3.2 | 1.2 | 0.74 | **0.05** | 0.13 | 0.33 | 0.14 |
| IGHV3-9 | 1.5 | 11 | 2.6 | 7.9 | 2.1 | 2 | 3.4 | 1.9 | 5.3 | 1.9 | 1.00 | 0.75 | 0.82 | 0.34 | 0.65 |
| IGHV4-28 | 0 | 0 | 1.5 | 1 | 2.1 | 0 | 0 | 0 | 0 | 0 | - | - | - | - | - |
| IGHV4-30-2 | 0 | 0 | 0 | 0 | 0 | 0 | 3 | 5 | 0 | 5.3 | - | - | - | - | - |
| IGHV4-30-4 | 1.5 | 7.8 | 2 | 5 | 1.6 | 3 | 3 | 1.7 | 1.5 | 1.8 | 1.00 | 1.00 | 0.06 | 1.00 | 0.84 |
| IGHV4-31 | 1.2 | 11 | 2.8 | 7.5 | 2.6 | 2.5 | 3 | 1.5 | 1.7 | 3.2 | 0.83 | 0.94 | 0.91 | 0.54 | 0.33 |
| IGHV4-34 | 0 | 0 | 3 | 1.5 | 4 | 0 | 5 | 2 | 0 | 1.7 | - | 1.00 | 0.50 | 1.00 | - |
| IGHV4-38-2 | 1 | 0 | 1 | 3 | 1.7 | 0 | 1 | 0 | 3 | 0.8 | - | 0.61 | 0.81 | 0.76 | 1.00 |
| IGHV4-39 | 1.3 | 5.9 | 2 | 6.4 | 2.1 | 1.2 | 0 | 0 | 0 | 12.3 | 1.00 | 0.11 | **0.0008** | 0.71 | 0.79 |
| IGHV4-4 | 1.7 | 2.1 | 1.1 | 9.7 | 1.6 | 3.2 | 5.3 | 2.5 | 10 | 2.7 | 0.59 | 0.94 | 0.30 | 0.54 | 1.00 |
| IGHV4-59 | 0.9 | 10.3 | 1.9 | 7 | 2.1 | 1.7 | 7 | 1.6 | 4 | 1.6 | 0.76 | 0.95 | 0.47 | 1.00 | 0.65 |
| IGHV4-61 | 0.5 | 5.5 | 1.4 | 2.4 | 1.7 | 0.3 | 4.3 | 1.3 | 8 | 4.3 | 1.00 | 0.86 | 0.12 | 1.00 | 0.54 |
| IGHV5-10-1 | 2 | 12 | 4.3 | 17 | 3 | 2.2 | 0 | 1.5 | 1.5 | 3.8 | 1.00 | 0.68 | 0.97 | 1.00 | 0.20 |
| IGHV5-51 | 2.3 | 2.5 | 1.9 | 3.9 | 2.6 | 0 | 0 | 4 | 2.6 | 1.5 | 0.06 | 0.62 | 0.15 | **0.03** | 0.77 |
| IGHV6-1 | 1.3 | 1.8 | 1.3 | 4 | 1.8 | 0 | 0 | 0 | 0 | 0 | - | - | - | - | - |
| IGHV7-4-1 | 0 | 0.5 | 1.5 | 0 | 3.5 | 0 | 0 | 0 | 0 | 0 | - | - | - | - | - |

**Supplemental Table 11.** Examples of new N-glycosylation positions in IGHV genes of 38 MM patients with IgG and IgA isotypes.

| IGHV gene |  | VH FR1 | VH CDR1 | VH FR2 | VH CDR2 | VH FR3 |
| --- | --- | --- | --- | --- | --- | --- |
| IGHV1-18 | N-glyc position | - | - | - | - | 66-69 |
|  | germline | - | - | - | - | NYAQ...AR |
|  | IgG patient 1 | - | - | - | - | NNTY. . . . . |
| IGHV1-2 | N-glyc position | 13-15 | - | - | - | 73-76 |
|  | germline | QV..KVSC.AS | - | - | - | NY.DTSI..AR |
|  | IgA patient 1 | . . . .NISC. . . | - | - | - | . . . . . . . . . . . |
|  | IgG patient 2 | . . . . . . . . . . . | - | - | - | . . . NSSI. . . . |
| IGHV1-69 | N-glyc position | - | - | - | - | 96-99 |
|  | germline | - | - | - | - | NY..EDTA.AR |
|  | IgG patient 3 | - | - | - | - | NY..NDTA.AR |
|  | IgG patient 4 | - | - | - | - | NY..NDTA.AR |
|  | IgG patient 5 | - | - | - | - | NY..NDTA.AR |
| IGHV1-8 | N-glyc position | - | - | - | 57-60 | - |
|  | germline | - | - | - | MNPNS..NT | - |
|  | IgA patient 2 | - | - | - | . NPTS. . . . | - |
|  | IgG patient 6 | - | - | - | . NPTS. . . . | - |
| IGHV2-26 | N-glyc position | - | - | - | - | 68-72 |
|  | germline | - | - | - | - | SYSTSL...AR |
|  | IgG patient 7 | - | - | - | - | SYNSSL. . . . . |
|  | IgG patient 8 | - | - | - | - | SYNSSL. . . . . |
| IGHV2-5 | N-glyc position | 13-15 | 30-32 | - | - | 68-72 |
|  | germline | QI...KPT...FS | GF...STS.VG | - | - | RYSPSLK...HR |
|  | IgA patient 3 | . . . . . . . . . . . . . | . . . . . . . . . . . | - | - | . . NPSL. . . . . . |
|  | IgA patient 4 | . . . . . . . . . . . . . | . . . . . . . . . . . | - | - | . . NPSL. . . . . . |
|  | IgA patient 5 | . . . . . . . . . . . . . | . . . . . NNS. . . | - | - | . . . . . . . . . . . . |
|  | IgG patient 9 | . . . . . NPT. . . . . | . . . . . . . . . . . | - | - | . . . . . . . . . . . . |
| IGHV2-70 | N-glyc position | - | - | - | - | 68-72 |
|  | germline | - | - | - | - | YYSTSL...AR |
|  | IgG patient 10 | - | - | - | - | . . NTSL. . . . . |
| IGHV3-11 | N-glyc position | - | 29-32 | - | - | - |
|  | germline | - | GF.TFSD..YY | - | - | - |
|  | IgG patient 11 | - | . . . NFSD. . . . | - | - | - |
| IGHV3-21 | N-glyc position | - | 35-38 | - | - | - |
|  | germline | - | GF... SSYS | - | - | - |
|  | IgG patient 12 | - | . . . . .NRSA | - | - | - |
| IGHV3-23 | N-glyc position | - | - | - | - | 81-86 |
|  | germline | - | - | - | - | YY..NSKNTL.AR |
|  | IgG patient 13 | - | - | - | - | . . . .NSSN . . . . . |
|  | IgG patient 14 | - | - | - | - | . . . . . . NNTL. . . |
| IGHV3-30 | N-glyc position | - | - | - | - | 77-87 |
|  | germline | - | - | - | - | YY..TISR.KNTL.AK |
|  | IgA patient 6 | - | - | - | - | . . . .NISR . . . . . . . . |
|  | IgG patient 15 | - | - | - | - | . . . . . . . . .NNTL. . . |
|  | IgG patient 16 | - | - | - | - | . . . . . . . . .NNTL. . . |
|  | IgG patient 17 | - | - | - | - | . . . . . . . . .NNTL. . . |
| IGHV3-30-3 | N-glyc position | - | - | - | - | 83-86 |
|  | germline | - | - | - | - | YY..KNTL.....AR |
|  | IgG patient 18 | - | - | - | - | YY..NNTL.....AR |
|  | IgG patient 19 | - | - | - | - | YY..NNTL.....AR |
|  | IgG patient 20 | - | - | - | - | YY..NPTN.....AR |
| IGHV4-30-4 | N-glyc position | - | - | - | - | 89-92 |
|  | germline | - | - | - | - | YYNPSL..KLSS.AR |
|  | IgG patient 21 | - | - | - | - | . . . . . . . .NMSS. . . |
|  | IgG patient 22 | - | - | - | - | . . . . . . . .NLTS. . . |
| IGHV4-39 | N-glyc position | - | - | - | - | 68-72 \| 81-83 |
|  | germline | - | - | - | - | Y.NPSL....SLKL...AR |
|  | IgG patient 23 | - | - | - | - | . .NPSL....NLTA... . . |
|  | IgG patient 24 | - | - | - | - | . .NPSL....NLSS... . . |
| IGHV4-4 | N-glyc position | - | - | - | 55-57. | 68-72 \| 82-84 |
|  | germline | - | - | - | IY...SGST | NYNPSLK.KLS..AR |
|  | IgA patient 7 | - | - | - | . . . . . . . . . | . .NPSL. .NLT.. . . |
|  | IgG patient 25 | - | - | - | . . . . .NNS. | . .NPSL. . . . . . . . . |
| IGHV4-61 | N-glyc position | - | - | - | - | 61-63 |
|  | germline | - | - | - | - | NY.NPSL..AR |
|  | IgG patient 26 | - | - | - | - | . . . NKSL. . . . |
| IGHV5-10-1 | N-glyc position | 20-23 | - | - | - | 68-72 |
|  | germline | EV.RISC.GS | - | - | - | NY....SPSF...AR |
|  | IgA patient 8 | EV.NISC.GS | - | - | - | . . . . . . . . . . . . . . . |
|  | IgG patient 27 | . . . . . . . . . . | - | - | - | . . . . . .NPSF. . . . . |
|  | IgG patient 28 | . . . . . . . . . . | - | - | - | . . . . . .NPSF. . . . . |
|  | IgG patient 29 | . . . . . . . . . . | - | - | - | . . . . . .NPSF. . . . . |
| IGHV5-51 | N-glyc position | - | - | - | - | 68-72 |
|  | germline | - | - | - | - | RY...SPSF...AR |
|  | IgG patient 30 | - | - | - | - | . . . . .NPSF. . . . . |

**Supplemental Table 12.** Examples removal of germline encoded positions in IGHV genes of 18 MM patients with IgG and IgA isotypes.

| IGHV gene |  | VH FR1 | VH CDR1 | VH FR2 | VH CDR2 | VH FR3 |
| --- | --- | --- | --- | --- | --- | --- |
| IGHV1-46 | N-glyc position | - | - | - | 58-60 | - |
|  | germline | - | - | - | INPS...ST | - |
|  | IgA patient 9 | - | - | - | .KPS. . . . . | - |
|  | IgG patient 31 | - | - | - | .KPS. . . . . | - |
|  | IgG patient 32 | - | - | - | .NKS. . . . . | - |
|  | IgG patient 33 | - | - | - | .KPS. . . . . | - |
| IGHV4-30-4 | N-glyc position | - | - | - | - | 68-71 |
|  | germline | - | - | - | - | YYNPSL . . . AR |
|  | IgG patient 34 | - | - | - | - | . . KPSL . . . . . . |
|  | IgG patient 35 | - | - | - | - | . . KPSL . . . . . . |
| IGHV4-34 | N-glyc position | - | - | - | - | 68-72 |
|  | germline | - | - | - | - | NYNPSL . . . AR |
|  | IgA patient 10 | - | - | - | - | . . KPSL . . . . . . |
| IGHV4-39 | N-glyc position | - | - | - | - | 68-72 |
|  | germline | - | - | - | - | Y.NPSL...AR |
|  | IgA patient 11 | - | - | - | - | . .NKSL. . . . . |
|  | IgA patient 12 | - | - | - | - | . .NKSL. . . . . |
|  | IgG patient 36 | - | - | - | - | . .NKSL. . . . . |
|  | IgG patient 37 | - | - | - | - | . .NPLL. . . . . |
|  | IgG patient 38 | - | - | - | - | . .NKSL. . . . . |
| IGHV4-4 | N-glyc position | - | - | - | - | 68-72 |
|  | germline | - | - | - | - | NYNPSL...AR |
|  | IgA patient 13 | - | - | - | - | . . SPSL. . . . . |
|  | IgG patient 39 | - | - | - | - | . . SPSL. . . . . |
|  | IgG patient 40 | - | - | - | - | . . NKSL. . . . . |
| IGHV4-59 | N-glyc position | - | - | - | - | 68-72 |
|  | germline | - | - | - | - | NYNPSL...AR |
|  | IgA patient 14 | - | - | - | - | NYNKSL...AR |
|  | IgA patient 15 | - | - | - | - | NYNKSL...AR |
| IGHV4-61 | N-glyc position | - | - | - | - | 61-63 |
|  | germline | - | - | - | - | NY.NPSL..AR |
|  | IgG patient 41 | - | - | - | - | . . .NKSL. . . . |
